# Overcoming Resistance in Triple-Negative Breast Cancer: A Translational Perspective on Next-Generation DNA Damage Response Inhibitors and Synthetic Lethality

**DOI:** 10.3390/molecules31132303

**Published:** 2026-07-01

**Authors:** Jakub Jończyk, Anna Czopek, Ulyana Kvinta, Aleksandra Skok, Agnieszka Zagórska

**Affiliations:** 1 Department of Medicinal Chemistry, Jagiellonian University Medical College, Medyczna 9, 30-688 Kraków, Poland; jakub.jonczyk@uj.edu.pl (J.J.); anna.czopek@uj.edu.pl (A.C.); 2 Students Scientific Group of Drug Discovery, Jagiellonian University Medical College, Św. Anny 12, 31-008 Kraków, Poland; ulyana.nosavich@student.uj.edu.pl (U.K.); aleksandra.skok@student.uj.edu.pl (A.S.)

**Keywords:** BRCA1/2, triple-negative breast cancer, poly(ADP-ribose) polymerase, synthetic lethality, RAD51, POLQ, ATR/CHK1, WEE1, homologous recombination deficiency

## Abstract

Triple-negative breast cancer (TNBC), particularly when associated with breast cancer susceptibility gene 1/2 (BRCA1/2) alterations or homologous recombination deficiency (HRD), remains therapeutically challenging because DNA repair vulnerabilities coexist with molecular heterogeneity, resistance, and toxicity constraints. This narrative review synthesizes mechanistic, preclinical, clinical, and translational evidence on DNA damage response (DDR)-targeted and synthetic lethality-based strategies in TNBC. We summarize TNBC biological heterogeneity, current biomarker-guided treatment options, mechanisms of poly(ADP-ribose) polymerase (PARP) inhibition and resistance, and emerging DDR targets, including ataxia telangiectasia and Rad3-related/checkpoint kinase 1 (ATR/CHK1), WEE1, DNA-dependent protein kinase (DNA-PK), RAD51, DNA polymerase theta (POLQ), neddylation-related pathways, and targeted protein degradation. The review highlights that PARP inhibitors and platinum agents provide clinically validated examples of exploiting HRD in selected populations, whereas most next-generation DDR inhibitors remain preclinical, investigational, or in early clinical trials. Resistance mechanisms, including BRCA reversion, homologous recombination restoration, replication fork stabilization, and checkpoint adaptation, limit durable benefit. Safety, target selectivity, overlapping toxicities, and the lack of standardized functional biomarkers further constrain translation. Future progress will require prospective biomarker validation, dynamic HRD assessment, rational scheduling of combinations, and medicinal chemistry approaches that improve therapeutic index rather than a broad application of DDR inhibition across all TNBC.

## 1. Introduction

Cancer is one of the leading causes of death and disease globally, second only to cardiovascular disease, imposing a substantial and growing burden on healthcare systems. According to the World Health Organization (WHO), cancer accounted for nearly 10 million deaths in 2020, corresponding to approximately one in six deaths globally. WHO and the International Agency for Research on Cancer (IARC) further estimate that the annual number of new cancer cases may exceed 35 million by 2050, representing an increase of about 77% compared with 2022, largely due to population ageing, population growth, and continued exposure to modifiable risk factors [1]. In 2022, the most common cancers worldwide (share of new cases) were lung cancer (12.4%), breast cancer (11.6%), colorectal cancer (9.6%), prostate cancer (7.3%), stomach cancer (4.9%).

Breast cancer is a heterogeneous disease that can be classified using several complementary approaches, including histopathological, immunohistochemical, and molecular classifications [2,3]. Histopathological classification is based on tumor morphology and tissue of origin. In contrast, immunohistochemical classification relies on the assessment of estrogen receptor (ER), progesterone receptor (PR), and human epidermal growth factor receptor 2 (HER2) and Ki-67 expression, which remains the foundation of routine clinical decision-making. The advent of gene expression profiling has further refined breast cancer classification by identifying intrinsic molecular subtypes with distinct biological characteristics, prognostic implications, and therapeutic sensitivities. Classification according to HER2 expression has recently gained significant clinical importance due to the development of novel HER2-targeted antibody-drug conjugates (ADCs). In addition to the traditional distinction between HER2-positive and HER2-negative breast cancer tumors are now further categorized as HER2-low, HER2-ultralow, or HER2-zero based on immunohistochemical (IHC) and in situ hybridization (ISH) findings [4]. This refined classification has emerged following the clinical success of trastuzumab deruxtecan [5], which demonstrated substantial benefit in patients with HER2-low breast cancer. Furthermore, ongoing clinical studies are evaluating additional HER2-directed ADCs, including trastuzumab duocarmazine (SYD985) [6] and disitamab vedotin (RC48) [7], in tumors with low levels of HER2 expression. Consequently, accurate assessment of HER2 status has become increasingly important for identifying patients who may benefit from these emerging targeted therapies [8].

Given the central role of homologous recombination deficiency (HRD) in TNBC biology, this review focuses on the therapeutic opportunities arising from the interplay between HRD, synthetic lethality, and DNA damage response (DDR)-targeted therapies. We discuss the molecular basis of these vulnerabilities, mechanisms of resistance that limit the efficacy of current PARP inhibitors (PARPi), and emerging approaches aimed at overcoming resistance and expanding treatment options for patients with BRCA1/2-mutant and HRD-positive TNBC.

## 2. Triple-Negative Breast Cancer Biology and Therapeutic Vulnerabilities

TNBC is not a single biological entity, but a clinically and molecularly heterogeneous group of tumors. Although grouping these tumors under a common clinical definition is useful, this definition does not capture the biological diversity that shapes prognosis, metastatic behavior, treatment response, and resistance. The biological heterogeneity of TNBC has been established through multiple molecular subtyping studies. These have identified basal-like, mesenchymal, immunomodulatory, and luminal androgen receptor-related subtypes, with subsequent work prioritizing tumor-intrinsic classifications such as basal-like 1, basal-like 2, mesenchymal, and luminal androgen receptor phenotypes [9,10,11].

TNBC subgroups differ in DNA damage repair capacity, growth factor signaling, immune activity, epithelial–mesenchymal transition (EMT), and androgen receptor dependence [12]. These differences provide a rationale for biomarker-driven treatment strategies rather than a uniform therapeutic approach to all TNBC cases. Table 1 summarizes major molecularly defined TNBC phenotypes and their potential therapeutic implications. Importantly, all TNBC therapeutic vulnerabilities should be interpreted according to their translational maturity. Some vulnerabilities correspond to clinically actionable settings, whereas others remain investigational or biologically plausible but insufficiently validated. Nonetheless, some remain in an investigational phase or are merely biologically plausible, and their therapeutic efficacy is yet to be substantiated through extensive, rigorous research.

Basal-like features are commonly observed in TNBC tumors and are associated with rapid cell proliferation, marked genomic instability, and frequent mutations in the TP53 tumor suppressor gene, which lead to loss of p53 tumor suppressor function [13,14]. This biological aspect partially explains why many TNBC tumors exhibit aggressive clinical behavior.

TNBC is also linked to defects in DNA repair mechanisms, particularly in the homologous recombination pathway involving BRCA1 and BRCA2 genes [15]. These defects may increase sensitivity to DNA-damaging agents, including platinum-based chemotherapy, and to synthetic lethality-based strategies such as PARP inhibition. Nevertheless, sensitivity to platinum agents or PARP inhibitors should not be inferred from TNBC status alone. It is most biologically and clinically justified in tumors with germline or somatic BRCA1/2 alterations, HRD-positive biology, or selected DDR defects, whereas HR-proficient TNBC may require different therapeutic rationales [16,17].

The tumor microenvironment has a major influence on TNBC progression and treatment response. Compared with other breast cancer subtypes, TNBC often contains higher numbers of tumor-infiltrating lymphocytes (TILs), reflecting its relatively high immunogenicity [18,19,20]. This has led to the successful introduction of immunotherapy, especially immune checkpoint inhibitors targeting the programmed death PD-1/programmed death-ligand 1 (PD-L1) pathway, in selected patients. TIL abundance is generally associated with a more favorable prognosis in early TNBC, but its predictive value depends on treatment context and should be distinguished from PD-L1-based selection used in some immunotherapy settings. In addition to lymphocytic infiltration, cancer-associated fibroblasts, macrophages, and inflammatory mediators can support tumor growth, invasion, immune escape, and resistance to therapy [21,22].

In a subset of cases, TNBC is also characterized by the presence of cancer stem-like cell features and activation of EMT, a process that enhances cell motility, invasiveness, metastatic dissemination, and resistance to systemic therapy [23]. Several signaling pathways, including PI3K/AKT/mTOR, EGFR, MYC, and TGF-β, are frequently dysregulated and play important roles in TNBC development and progression [24,25]. Although these pathways provide biologically plausible therapeutic vulnerabilities, their clinical maturity remains limited. In contrast to established or clinically actionable biomarkers such as BRCA1/2 alterations, PD-L1 expression, TROP-2 expression, or HER2-low status, targeting PI3K/AKT/mTOR, EGFR, MYC, or TGF-β in TNBC remains investigational, context-dependent, or supported by limited clinical benefit [26].

### Current Clinical Treatment Landscape in TNBC

For many patients with stage II–III early TNBC who are eligible for neoadjuvant systemic therapy, treatment commonly includes anthracycline- and taxane-based chemotherapy, often with carboplatin, with pembrolizumab integrated in selected guideline-defined settings. After completion of systemic therapy, patients undergo surgery, which may be followed by radiotherapy depending on the clinical presentation.

Patients who fail to achieve a pathological complete response (non-pCR) after neoadjuvant treatment may receive additional adjuvant therapy. For carriers of germline BRCA mutations, olaparib is recommended, whereas capecitabine may be considered for patients with residual disease after surgery. In cases of disease recurrence, treatment selection is based on molecular characteristics and previous therapies. In metastatic TNBC, immune checkpoint inhibitor, atezolizumab use depends on PD-L1 status, treatment line, regulatory region, and applicable guideline recommendations. For patients harboring BRCA mutations, the PARP inhibitors olaparib or talazoparib represent additional therapeutic options. Chemotherapy remains an alternative first-line approach when targeted therapies or immunotherapy are not indicated. Following disease progression, sacituzumab govitecan is the preferred second-line treatment option. In subsequent lines of therapy, conventional chemotherapy may be administered according to prior treatment exposure, patient condition, and tumor characteristics. Advances in molecular profiling have revealed distinct TNBC subtypes, which improved understanding of TNBC biology and paved the way for more personalized therapeutic strategies and the identification of novel molecular targets (Table 2). However, despite recent advances in immunotherapy, PARP inhibitors, and antibody-drug conjugates, treatment options for patients with advanced TNBC remain limited, and therapeutic resistance continues to represent a major clinical challenge.

## 3. Mechanism of Action of DDR-Targeted and Synthetic Lethality-Based Strategies in TNBC

**Figure 1:**
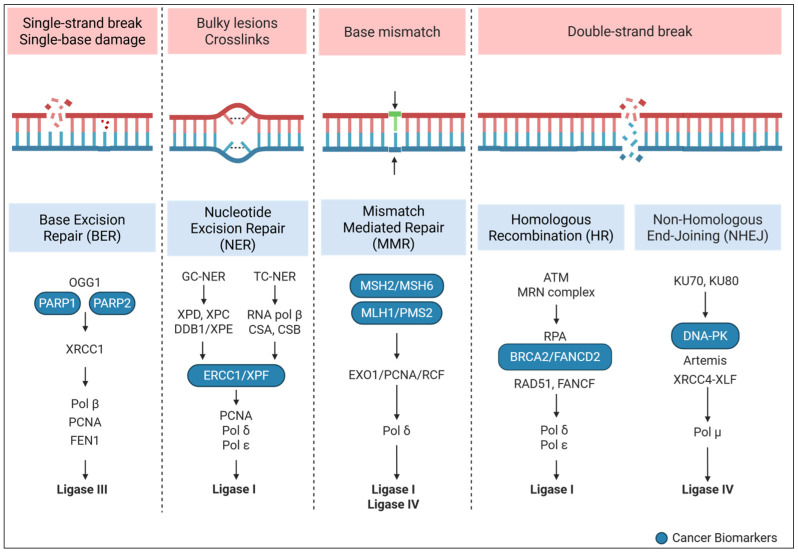
Different types of DNA lesions are repaired by distinct DNA damage response (DDR) mechanisms. Single-base damage and single-strand breaks are primarily repaired through the base excision repair (BER) pathway involving PARP1/2. Bulky DNA lesions and DNA crosslinks are repaired by nucleotide excision repair (NER), whereas base mismatches are corrected by the mismatch repair (MMR) system. DNA double-strand breaks are repaired either by the high-fidelity homologous recombination (HR) pathway, involving BRCA2, FANCD2, RAD51, and ATM signaling, or by the error-prone non-homologous end joining (NHEJ) pathway mediated by DNA-PK and associated repair factors. Proteins highlighted in blue represent clinically relevant biomarkers or therapeutic targets currently investigated in precision oncology. Created in BioRender. Zagórska, A. (2026) https://BioRender.com/gxi11e6 (accessed on 16 June 2026).

The DDR system comprises a complex network of signaling pathways that detect DNA lesions, halt cell-cycle progression, and coordinate DNA repair. Several repair mechanisms are involved in maintaining genomic integrity, including base excision repair (BER), nucleotide excision repair (NER), mismatch repair (MMR), homologous recombination repair (HRR), and non-homologous end joining (NHEJ). Among these pathways, HRR is particularly relevant to BRCA1/2-mutant and HRD-enriched TNBC because it provides high-fidelity repair of DNA double-strand breaks (DSBs) and replication-associated lesions during the S and G2 phases of the cell cycle. Figure 1 summarizes the main types of DNA lesions and the corresponding DDR repair pathways. Key HRR proteins include BRCA1, BRCA2, PALB2, and RAD51, and impairment of this pathway contributes to genomic instability, replication stress, and therapeutic vulnerability in selected TNBC subgroups [27,28]. HRR impairment should therefore be interpreted as a biomarker-defined vulnerability within BRCA1/2-mutant or HRD-enriched TNBC rather than as a universal feature of all TNBC tumors [29].

### 3.1. Synthetic Lethality as a Therapeutic Principle in HRD-Enriched TNBC

Synthetic lethality refers to a genetic or functional interaction in which the disruption of either of two genes or pathways alone is compatible with cell survival, whereas their simultaneous impairment leads to cell death [30,31]. In cancer therapy, this concept is exploited by targeting a DDR pathway that becomes essential in tumor cells already carrying a defect in another repair mechanism. The most successful example of synthetic lethality in TNBC involves the inhibition of PARP in tumors with BRCA1 or BRCA2 mutations, where impaired homologous recombination increases dependence on PARP-mediated repair and replication stress tolerance [32].

### 3.2. BRCA1 and BRCA2 Deficiency as the Mechanistic Basis of HRD

The effectiveness of therapeutic strategies that exploit BRCA deficiency is rooted in the fundamental roles of BRCA1 and BRCA2 in homologous recombination, the protection of replication forks, and the coordination of cell-cycle checkpoints [33,34]. BRCA1 acts primarily upstream in HRR by promoting DNA end resection, regulating repair pathway choice, and coordinating checkpoint signaling through interactions with ATM, ATR, and downstream repair factors. BRCA2 acts downstream by controlling RAD51 loading onto single-stranded DNA, thereby enabling RAD51 nucleofilament formation, homology search, strand invasion, and accurate repair.

**Figure 2:**
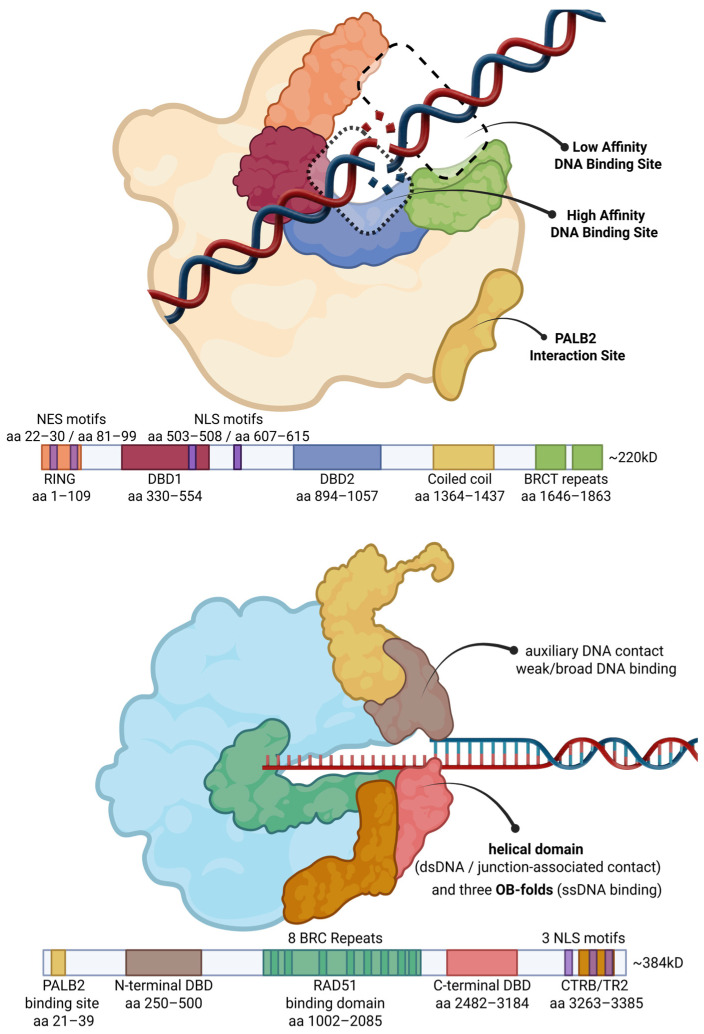
Domain organization and trafficking motifs of human BRCA1 (**top**) and BRCA2 (**bottom**). The structural architecture of these proteins governs DNA damage recognition, checkpoint signaling, and HRR factor recruitment. **Top**: BRCA1 domains include RING (salmon), DBD1 (red), DBD2 (blue), coiled-coil (yellow), and BRCT (green). **Bottom**: BRCA2 features the PALB2-binding site (yellow), N-terminal DBD (brown), RAD51-binding domain, C-terminal DBD (salmon), and CTRB/TR2 domains (orange). Purple boxes denote nuclear localization (NLS) and export (NES) signals across both sequences. (2026) Created in BioRender. Jończyk, J. (2026) https://BioRender.com/idldolo (accessed on 16 June 2026).

Beyond canonical DSB repair, BRCA1/2 also preserve genome stability by protecting stalled or reversed replication forks from nucleolytic degradation, a function that influences both therapeutic sensitivity and acquired resistance to DNA-damaging agents [35,36]. Loss of BRCA1/2 function therefore promotes genomic instability, replication stress, and increased reliance on compensatory DNA repair and checkpoint pathways. This creates a biological context in which inhibition of PARP and selected replication-stress response pathways can selectively impair tumor cell survival. Figure 2 illustrates the domain organization of BRCA1 and BRCA2.

### 3.3. PARP Inhibition: Catalytic Inhibition, PARP Trapping, and Replication Fork Collapse

**Figure 3:**
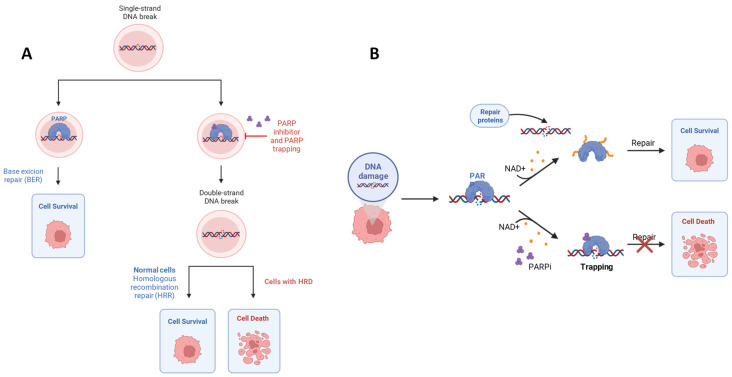
Mechanism of PARP Inhibitors in DNA Damage Repair (based on [42,43]). (**A**) DNA repair pathways and cellular outcomes: single-strand breaks are repaired by PARP-mediated base excision repair, while double-strand breaks are resolved by homologous recombination; HR-deficient cells fail to repair and undergo cell death. (**B**) Mechanism of PARP inhibition and PARP trapping. Created in BioRender. Czopek, A. (2026) https://BioRender.com/gxi11e6 (accessed on 16 June 2026).

PARP inhibitors exert their antitumor activity through two complementary mechanisms: catalytic inhibition and PARP trapping. By competing with NAD^+^, PARPi block the catalytic activity of PARP enzymes and prevent the synthesis of poly(ADP-ribose) (PAR) chains required for PARP1 auto-PARylation and dissociation from DNA [29,36]. As a result, PARP1 remains bound to sites of DNA damage, forming cytotoxic DNA-protein complexes that physically obstruct replication forks (Figure 3A) [37,38,39]. In HR-deficient cells, replication stress induced by PARP inhibition promotes replication fork stalling and collapse, leading to the accumulation of DNA double-strand breaks. Because these lesions cannot be efficiently repaired in the absence of functional HR, genomic instability progressively increases, ultimately resulting in tumor cell death (Figure 3) [40,41].

### 3.4. Mechanisms of Resistance to PARP Inhibitors in TNBC

The most clinically relevant mechanisms of resistance to PARP inhibitors in TNBC include BRCA1/2 reversion mutations, restoration of homologous recombination proficiency, replication fork stabilization, overexpression of drug efflux transporters, and activation of DNA damage response pathways. Among these, secondary BRCA1/2 mutations are of particular importance, as they restore the function of BRCA proteins and reestablish the ability of tumor cells to repair DNA double-strand breaks through homologous recombination. Consequently, the synthetic lethality underlying the therapeutic efficacy of PARP inhibitors is lost [16,44].

Another important mechanism of resistance involves the restoration of homologous recombination independently of direct BRCA function recovery. This process may occur through increased expression of RAD51, loss of 53BP1, or alterations in the Shieldin complex, thereby enabling efficient DNA repair despite the presence of BRCA deficiencies [45]. Increasing evidence also highlights the role of replication fork stabilization. Cancer cells may acquire the ability to protect stalled replication forks from nuclease-mediated degradation, reducing the accumulation of DNA damage and diminishing sensitivity to PARP inhibition [46].

Overexpression of the ABCB1 transporter (P-glycoprotein, MDR1), which actively exports drugs from cancer cells, represents another mechanism of resistance. This leads to reduced intracellular concentrations of PARP inhibitors and consequently decreases their antitumor activity [45]. In addition, activation of the ATR-CHK1-WEE1 signaling axis plays a significant role in mediating resistance. As a major regulator of the cellular response to replication stress and DNA damage, this pathway enables tumor cells to survive PARP inhibition by enhancing cell-cycle checkpoint control and DNA repair mechanisms [45,46].

**Figure 4:**
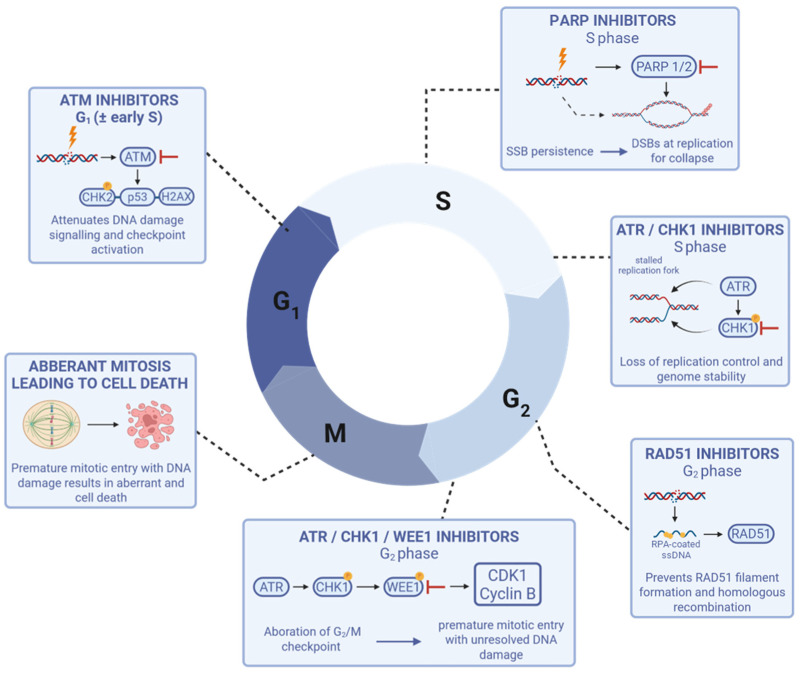
Cell cycle phase—specific activity of DNA damage response inhibitors. The schematic shows the cell cycle (G1, S, G2, M) and highlights where major DDR inhibitors act. In G1, ATM inhibitors disrupt early DNA damage signaling and checkpoint activation. In the S phase, PARP inhibitors cause accumulation of single-strand breaks that convert into double-strand breaks, while ATR/CHK1 inhibitors impair replication stress responses, leading to genomic instability. In G2, RAD51 inhibitors block homologous recombination, and ATR/CHK1/WEE1 inhibitors override the G2/M checkpoint, promoting premature mitotic entry despite DNA damage. Cells entering M phase with unresolved damage undergo aberrant mitosis and cell death. Created in BioRender. Czopek, A. (2026) https://BioRender.com/gxi11e6 (accessed on 16 June 2026).

These resistance mechanisms are currently the primary targets of advanced combination treatment strategies in TNBC. Ongoing research focuses on combining PARP inhibitors with ATR, CHK1, or WEE1 inhibitors, as well as with agents targeting DNA repair pathways and genomic stability. Consequently, many DDR-targeted strategies, including ATR, WEE1, DNA-PK, and POLQ inhibition, should currently be regarded as translationally promising rather than clinically validated approaches (Figure 4).

## 4. Preclinical Evidence for DDR-Targeted Strategies

Current drug discovery efforts increasingly focus on next-generation DDR-targeted strategies that may enhance, extend, or restore the therapeutic benefit of PARP inhibitor-based approaches [47]. Importantly, these approaches are primarily being investigated as strategies to overcome resistance to currently approved therapies rather than as direct replacements for standard treatment. Therefore, DDR-targeted therapeutic strategies aim to impair the cellular response to DNA damage through the inhibition of key DDR regulators, including ATR, CHK1, WEE1, DNA-PK, RAD51, and POLQ. By disrupting essential DNA repair mechanisms and cell-cycle checkpoint control, these approaches promote the accumulation of genomic damage and increase the susceptibility of cancer cells to chemotherapy, radiotherapy, PARP inhibitor-based treatments, or other DNA-damaging treatments. These strategies differ substantially in translational maturity, but most ATR/CHK1-, WEE1-, DNA-PK-, RAD51-, and POLQ-directed approaches should currently be regarded as preclinical, investigational, or early clinical strategies [46,48].

### 4.1. ATR/CHK1 Inhibition

ATR (Ataxia Telangiectasia and Rad3-related) is a central regulator of the cellular response to replication stress (RS) and DNA damage. When DNA replication is disrupted, ATR is recruited to replication protein A (RPA)-coated single-stranded DNA (ssDNA) at stalled replication forks, where it activates checkpoint kinase 1 (CHK1). The ATR-CHK1 axis stabilizes replication forks, suppresses excessive origin firing, slows cell-cycle progression, and coordinates DNA repair [49,50]. These functions are particularly relevant in HR-deficient or replication-stress-high TNBC models, where tumor cells may become dependent on checkpoint-mediated survival mechanisms. This dependency is expected to be strongest in molecular contexts characterized by HRD, high replication stress, ATM pathway defects, or impaired G1/S checkpoint control, rather than in TNBC as a uniform disease category.

Pharmacological inhibition of ATR or CHK1 disrupts these protective mechanisms, resulting in uncontrolled origin firing, depletion of nuclear RPA pools, replication fork collapse, and the accumulation of lethal DNA double-strand breaks (DSBs) [51,52]. In BRCA1/2-mutated and HR-deficient tumors, ATR/CHK1 inhibition may therefore induce synthetic lethal or synthetic lethal-like effects by disabling compensatory checkpoint pathways required for survival under conditions of persistent RS. Preclinical studies have demonstrated strong synergy between ATR or CHK1 inhibition and PARP inhibitors (PARPi). ATR blockade prevents the restoration of HR activity, as evidenced by reduced RAD51 foci formation, and overrides G2/M checkpoint control, thereby sensitizing tumor cells to PARPi even in resistant or replication-stress-dependent models [53,54,55].

**Figure 5:**
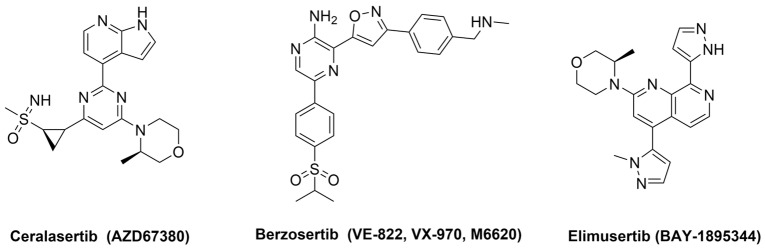
Representative small-molecule inhibitors targeting the ATR-CHK1.

Several ATR and CHK1 inhibitors have entered preclinical and early clinical development as potential strategies to exploit replication stress in TNBC and other homologous recombination-deficient tumors. Clinical development is advancing with potent molecules, including ceralasertib (AZD6738), berzosertib (VE-822, VX-970, M6620), and elimusertib (BAY-1895344), which are presently in Phase I/II trials [56,57,58] (Figure 5). The efficacy of these inhibitors is under assessment for use as standalone treatments and in conjunction with other therapies to enhance tumor responsiveness to platinum agents and radiotherapy [50,56]. Ultimately, ATR/CHK1 inhibition represents a strategy to convert replication stress tolerance into a lethal vulnerability by forcing premature mitotic entry with fragmented genomes [59].

In preclinical TNBC models, prexasertib impaired homologous recombination repair through suppression of BRCA1 and RAD51 expression, leading to a greater than 55% reduction in HR efficiency. Combination treatment with olaparib produced synergistic antitumor effects, resulting in increased DNA damage and enhanced cell death across multiple TNBC cell lines, supporting CHK1 inhibition as a strategy to sensitize TNBC to PARP inhibitors [60]. In TNBC patient-derived xenograft models, ceralasertib combined with olaparib produced marked antitumor activity, including complete regression in a BRCA2-mutant PDX model under defined experimental dosing conditions [55]. Significant antitumor activity was also observed when ceralasertib was combined with carboplatin or irinotecan, producing greater tumor growth inhibition than either agent alone [55].

Collectively, these findings provide strong preclinical support for ATR/CHK1 inhibition as a biologically plausible strategy for exploiting replication stress and PARP inhibitor vulnerability in selected TNBC models. Nevertheless, clinical evidence remains limited. Ongoing Phase I/II biomarker-defined clinical studies are expected to yield the evidence necessary to confirm their therapeutic value [21,46].

### 4.2. ATR/WEE1 Inhibition

**Figure 6:**
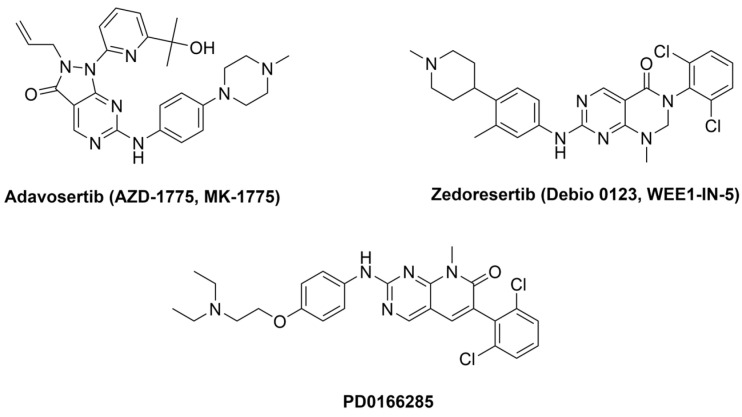
Representative small-molecule WEE1 inhibitors.

WEE1 kinase constitutes another regulator of the DDR network. Through inhibitory phosphorylation of CDK1 and CDK2 at Tyr15, WEE1 restrains S-phase progression and prevents premature mitotic entry, allowing sufficient time for DNA repair [52,53]. TNBC frequently harbors TP53 mutations and defective G1/S checkpoint control, making tumor cells highly dependent on WEE1-mediated regulation of the G2/M checkpoint. Consequently, inhibition of WEE1 using adavosertib (AZD1775, MK-1775), zedoresertib (Debio 0123), or PD0166285 (Figure 6) deregulates CDK activity, promotes unscheduled replication origin firing, depletes nucleotide pools, and ultimately induces mitotic catastrophe [50,61].

Preclinical studies suggest that WEE1 inhibition may impair RAD51 recruitment and induce an HRD-like state, potentially sensitizing HR-proficient tumors to PARP inhibition [62]. This strategy may be valuable in PARPi-resistant tumors or cancers exhibiting residual HR activity, where WEE1 inhibition forces cells to progress through mitosis despite extensive genomic damage.

Additional observations, including cGAS–STING activation [62] and PTEN-loss-associated sensitivity [63], remain model-dependent and should be interpreted as emerging hypotheses rather than established predictive biomarkers. PTEN deficiency may enhance vulnerability to ATR/WEE1 inhibition through increased replication stress, while cGAS–STING activation may reflect treatment-induced genomic instability and immune stimulation. Nevertheless, both associations require further clinical validation before being considered actionable biomarkers.

Although WEE1 inhibition has shown encouraging activity in preclinical models and early-phase clinical studies, its clinical utility in TNBC remains to be confirmed in larger randomized trials.

### 4.3. DNA-PK Targeting

DNA-dependent protein kinase (DNA-PK) is a central component of the non-homologous end joining (NHEJ) pathway and contributes to the repair of DNA double-strand breaks, particularly in contexts where HRR is impaired [50]. Inhibition of DNA-PK disrupts this compensatory mechanism, leading to the accumulation of unrepaired DNA damage, genomic instability, and ultimately cancer cell death [53]. In this context, DNA-PK inhibition is best viewed as a combination-oriented strategy designed to sensitize tumors to radiotherapy, chemotherapy, or other DDR-targeted agents, rather than as an established stand-alone therapeutic approach in TNBC.

Beyond its established role in NHEJ, DNA-PK also contributes to the cellular response to replication stress by supporting ATR-dependent checkpoint signaling at stalled replication forks and limiting excessive replication origin firing [64]. Emerging evidence further suggests a synthetic lethal interaction between ATM and DNA-PK, as ATM-deficient tumors may become particularly dependent on DNA-PK activity for survival. Consequently, DNA-PK inhibition represents a therapeutic strategy for selectively targeting ATM-deficient cancers and enhancing sensitivity to DNA-damaging treatments [30,65].

Recent studies have also highlighted the potential of combining DNA-PK inhibitors with epigenetic therapies. Histone deacetylase (HDAC) inhibitors can impair the expression of key DNA repair proteins, including RAD51 and 53BP1, thereby increasing tumor sensitivity to DNA-PK blockade [63]. Similarly, bromodomain-containing protein 4 (BRD4) inhibitors reduce CtIP expression and induce a homologous recombination-deficient (HRD) phenotype, creating a “BRCAness” state that further enhances susceptibility to both DNA-PK and PARP inhibition [66].

**Figure 7:**
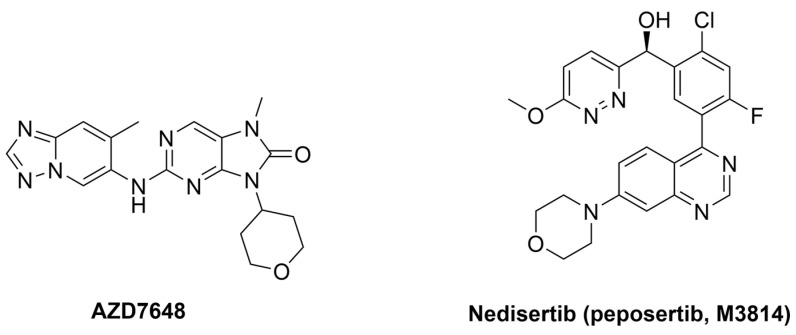
Representative small-molecule DNA-PK inhibitors.

The therapeutic rationale for targeting DNA-PK is therefore to eliminate a major compensatory DNA repair pathway and increase the vulnerability of tumor cells to genomic damage. Several selective DNA-PK inhibitors, including AZD7648 and peposertib (M3814) (Figure 7), are currently being evaluated in clinical trials, primarily in combination with radiotherapy, chemotherapy, and other DDR-targeted agents to overcome treatment resistance and improve therapeutic efficacy [50,66].

However, at present, the therapeutic potential of DNA-PK inhibition is supported primarily by preclinical evidence, and clinical validation in TNBC is still limited.

### 4.4. RAD51 Modulation

RAD51 recombinase serves as the central effector of HR, mediating critical strand invasion and homology search steps for the repair of DNA double-strand breaks [67,68]. RAD51 targeting is most relevant in resistance contexts in which tumor cells retain or restore HR activity despite upstream repair defects, for example, through RAD51 upregulation, 53BP1 loss, or other HR-restoring mechanisms. Accordingly, RAD51 modulation has been explored as a strategy to disrupt residual or restored homologous recombination activity in tumors exposed to PARP inhibitors or DNA-damaging therapy [68,69,70].

**Figure 8:**
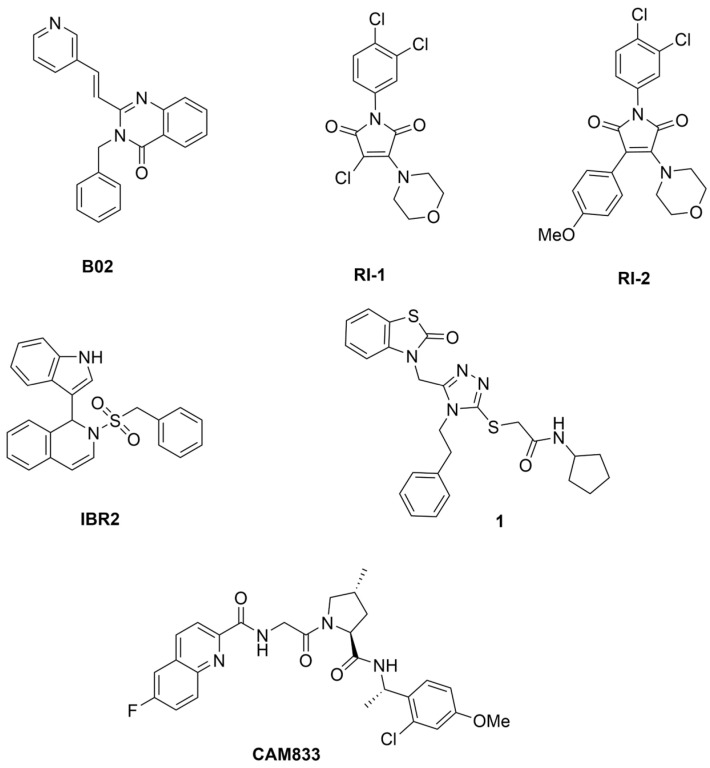
Representative approaches to RAD51 modulation, including direct RAD51 inhibitors, agents disrupting RAD51 multimerization or RAD51-BRCA2 interactions, and indirect modulators of RAD51 expression or activation.

Pharmacological strategies to modulate this target include direct small-molecule inhibitors like B02, which disrupts RAD51 binding to DNA, while RI-1 or RI-2 work by destabilizing the monomer-monomer interface crucial for nucleofilament formation [68,71] (Figure 8). Another class of agents, including IBR2 and its derivatives, disrupts RAD51’s ability to form multimers and accelerates its proteasome-mediated degradation. Therapeutic efforts also focus on the disruption of the RAD51-BRCA2 interaction using inhibitors such as CAM833, triazole derivatives, or computationally designed aptamers that compete with BRCA2 BRC repeats for binding pockets on RAD51 [70,72,73]. Emerging indirect methods include employing bromodomain and extra-terminal (BET) inhibitors to reduce RAD51 transcription or CHK1 inhibitors to block its post-translational activation [67,74].

**Figure 9:**
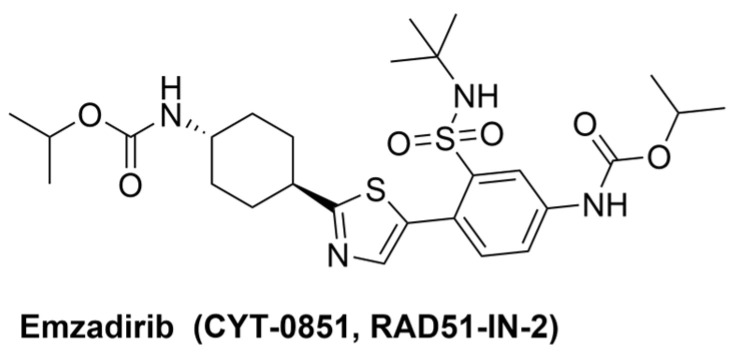
Emzadirib (RAD51-IN-2, CYT-0851).

Preclinical evidence demonstrates that RAD51 inhibition can reverse PARP inhibitor (PARPi) resistance, particularly in settings where resistance is driven by partial HR restoration, such as through Zinc finger protein 251 (ZNF251) haploinsufficiency or 53BP1 loss [75]. Some inhibitors, such as emzadirib (RAD51-IN-2, CYT-0851; Figure 9), have advanced to early-stage clinical trials (NCT03997968); however, in June 2023, the CYT-0851 program was discontinued due to failure to meet the criteria for proceeding to the next stage of clinical development. Nevertheless, the compelling biological rationale of RAD51-targeted therapies drives research into numerous other agents that are still in the preclinical or early translational stage, highlighting the complexity of targeting this essential protein [76].

### 4.5. POLQ/Polθ TMEJ/Alt-NHEJ as Target

DNA polymerase theta (Polθ), encoded by the POLQ gene, is a unique A-family polymerase and helicase fusion protein that serves as the essential effector of theta-mediated end joining (TMEJ), also known as alternative nonhomologous end-joining (alt-NHEJ) [77,78]. Given that the TMEJ pathway is characterized by its error-prone and inherently mutagenic nature, tumors with BRCA1/2 deficiency or HRD become dependent on this pathway to address resected double-strand breaks that the standard HR machinery cannot handle [79,80]. This dependency creates a selective vulnerability observed in preclinical HR-deficient models, rather than an established therapeutic window in patients.

Beyond double-strand break repair, Polθ has also been implicated in the suppression of single-stranded DNA gaps and protection of stressed replication forks, processes that may support survival in tumors with primary or acquired HR defects [77,78]. POLQ inhibition has therefore been investigated as a strategy to overcome PARP inhibitor resistance, particularly in settings where resistance involves HR rewiring mechanisms such as loss of 53BP1 or Shieldin complex alterations [80,81].

**Figure 10:**
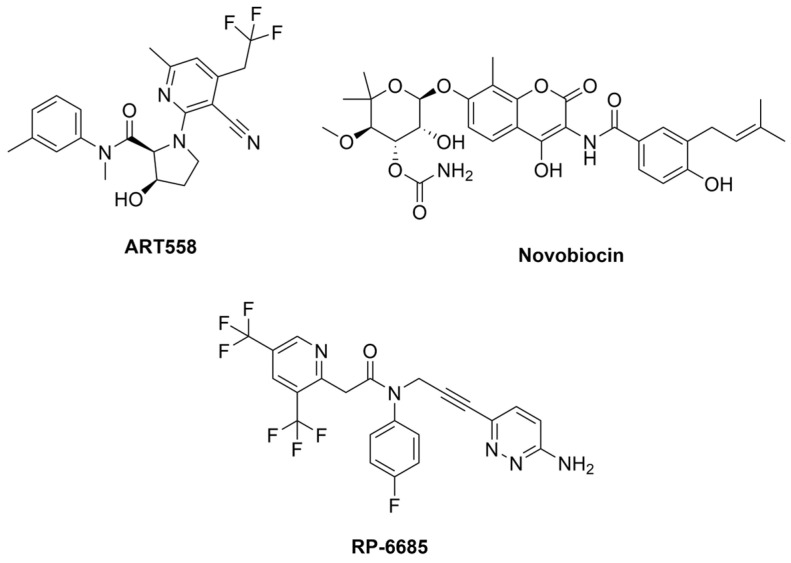
Representative small-molecule Polθ inhibitors.

Preclinical studies have evaluated several first-in-class small-molecule Polθ inhibitors, including ART558, novobiocin, and RP-6685, which target the ATPase or polymerase functions of the enzyme (Figure 10) [79]. Representative Polθ inhibitors differ in the enzymatic function they target, including ATPase/helicase-related activity and polymerase-domain activity. Current research is exploring whether POLQ expression or other HRD-associated features can serve as predictive biomarkers for sensitivity to these agents [82].

POLQ inhibition represents one of the biologically compelling emerging synthetic lethality strategies in BRCA-associated and HRD cancers. However, current evidence remains predominantly preclinical, and prospective clinical validation is required before POLQ inhibition can be considered an established therapeutic approach in TNBC.

## 5. Clinical Evidence

**Figure 11:**
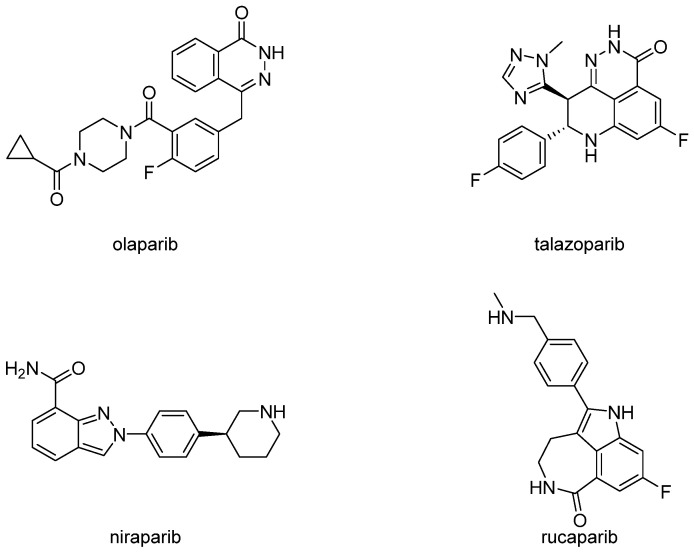
Structures of PARP inhibitors: olaparib, talazoparib, niraparib, and rucaparib.

Among approved PARP inhibitors across oncology, olaparib and talazoparib (Figure 11) are the clinically established agents for selected patients with germline BRCA1/2-mutated, HER2-negative breast cancer, including TNBC. While these drugs target the same molecular pathway, important differences in their pharmacological properties may influence treatment outcomes and clinical use [37,38,39,83]. Talazoparib is the most potent PARP trapper, demonstrating approximately 100-fold greater trapping potency than olaparib or rucaparib [37,39,83]. This increased potency allows treatment at much lower daily doses [39]. Pharmacokinetic characteristics also vary among these inhibitors: Talazoparib and niraparib have relatively long half-lives that enable once-daily dosing, whereas olaparib and rucaparib generally require twice-daily administration. Additionally, their metabolic pathways differ. Olaparib is primarily metabolized by cytochrome P450 3A4 isoform and is therefore susceptible to drug–drug interactions, while niraparib is metabolized mainly by carboxylesterases and talazoparib undergoes minimal hepatic metabolism, being largely excreted unchanged via the kidneys [39,83].

PARP inhibitors in TNBC have been evaluated across monotherapy and multiple combination strategies (Table 3). In monotherapy, the Phase 2 study (NCT00679783) assessed olaparib (400 mg twice daily) in 91 patients with advanced ovarian cancer or TNBC, including 26 breast cancer cases. No confirmed objective responses were observed in TNBC; however, disease control reached 70% in patients with BRCA mutations compared with 19% in those without mutations, with manageable toxicity consistent with the known safety profile of PARP inhibitors [84,85]. Another Phase 2 study (NCT02401347) evaluated talazoparib (1 mg daily) in metastatic HER2-negative or TNBC patients with non-BRCA homologous recombination mutations and included 21 participants, achieving an objective response rate (ORR) of 31% and a clinical benefit rate of 54%, with notable responses in PALB2-mutated tumors and a safety profile consistent with prior experience with talazoparib [86].

**Figure 12:**
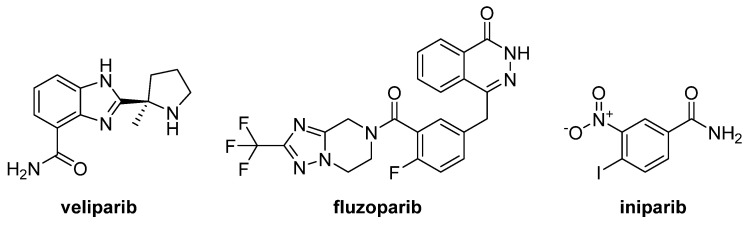
PARP inhibitors in clinical trials in TNBC.

**Figure 13:**
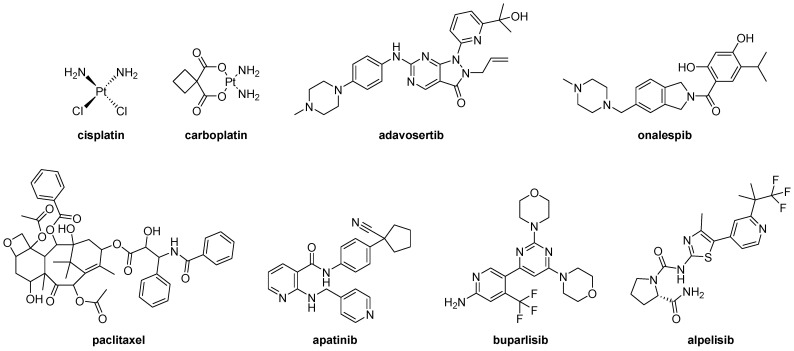
Structure of agents evaluated in combination with PARP inhibitors in TNBC in randomized clinical trials. Shown are cisplatin and carboplatin (DNA crosslinking platinum agents), paclitaxel (microtubule stabilizer inhibiting mitosis), onalespib (HSP90 inhibitor), apatinib (VEGFR2 inhibitor targeting angiogenesis), alpelisib (PI3Kα inhibitor), buparlisib (pan-PI3K inhibitor), and adavosertib (WEE1 kinase inhibitor).

In the randomized Phase 2 study (NCT02595905) involving 335 participants, veliparib (Figure 12, 300 mg twice daily, days 1–14) plus cisplatin (Figure 13) improved progression-free survival in BRCA-mutated and BRCA-like cohorts but increased hematologic toxicity, particularly anemia and thrombocytopenia [87]. In contrast, study NCT01074970 involving 128 participants found no significant improvement in 2-year disease-free survival with cisplatin (Figure 13) vs. cisplatin plus rucaparib, 100 mg weekly (54.2% vs. 64.1%), with no meaningful increase in toxicity, likely reflecting subtherapeutic dosing [88]. Similarly, a Phase 2 study (NCT01204125) involving 141 participants evaluated the addition of iniparib (Figure 12) to weekly paclitaxel (Figure 13) using two dosing schedules (once- and twice-weekly). No improvement in antitumor activity was observed, with similar pathologic complete response (pCR) rates across groups (approximately 19–22%; 21% with paclitaxel alone, 22% with once-weekly iniparib, and 19% with twice-weekly iniparib). Secondary endpoints were also comparable, and the regimen was generally well tolerated, aside from a slight increase in grade 3/4 neutropenia in the twice-weekly group, consistent with the lack of true PARP inhibition [89].

Targeted and immunotherapy combinations demonstrate more consistent activity. The Phase 1b study (NCT01623349) involving 17 participants combined alpelisib (Figure 13), a PI3K inhibitor, with olaparib, achieving an objective response rate of 18% and a clinical benefit rate of 35%, including responses in BRCA wild-type TNBC, suggesting [90] activity beyond BRCA-mutated disease, with manageable toxicity despite the addition of PI3K inhibition [91,92]. In the Phase 2 study (NCT02657889) involving 55 participants, niraparib (200 mg daily) plus pembrolizumab (200 mg every 3 weeks) achieved an objective response rate of 21% and a disease control rate of 49%, with higher efficacy in BRCA-mutated tumors (objective response rate 47%; progression-free survival 8.3 months), suggesting greater benefit in patients with BRCA mutations, and no unexpected safety signals beyond known class-specific toxicities [90]. In another Phase 2 study (NCT03167619) involving 45 participants, olaparib (300 mg twice daily) was administered alone or in combination with durvalumab (1500 mg every 4 weeks) as maintenance after platinum response, improving median progression-free survival (4.0 vs. 6.1 months) irrespective of BRCA status, with a tolerable safety profile and manageable immune-related adverse events [93].

Additional targeted approaches include antiangiogenic combinations. In Phase 1 study (NCT03075462) involving 52 women aged 18–70 from China, including 22 with TNBC, fluzoparib (40–100 mg twice daily) plus apatinib (Figure 12 and Figure 13), VEGFR-targeted tyrosine kinase inhibitor, (250–500 mg once daily; recommended phase 2 dose: 100 mg twice daily/500 mg once daily) achieved an objective response rate of 22.7% and a disease control rate of 54.5% in TNBC, with improved outcomes in BRCA-mutated patients (objective response rate 66.7%; progression-free survival 5.5 vs. 2.8 months), and a manageable but dose-dependent toxicity profile consistent with antiangiogenic therapy [94].

Early-phase studies further support the feasibility of diverse combinations. Phase 1 study (NCT03109080) involving 24 women evaluated olaparib (50–200 mg twice daily) with radiotherapy in high-risk TNBC, showing no dose-limiting toxicities, a 3-year overall survival of 83%, and an event-free survival of 65% [95,96]. Another Phase 1 study (NCT01445418) combined intravenous carboplatin (Figure 13, area under the curve 3–5) with oral olaparib (100–400 mg twice daily) in recurrent breast and ovarian cancers, identifying increased carboplatin hypersensitivity in BRCA mutation carriers, manageable with premedication [95,96]. In another Phase 1 trial (NCT02898207), olaparib was evaluated in combination with the HSP90 inhibitor onalespib (Figure 13) in 28 heavily pretreated patients with advanced solid tumors, including recurrent ovarian cancer and TNBC. The highest well-tolerated dose combinations were 300 mg twice daily olaparib with 40 mg/m^2^ *i.v*. onalespib and 200 mg twice daily olaparib with 80 mg/m^2^ *i.v*. onalespib, although a single maximum tolerated dose was not established. Despite no objective responses, 32% of patients achieved durable stable disease lasting at least 24 weeks, with acceptable tolerability and no new safety concerns [97].

The Phase 2 clinical trial (NCT03012477) evaluated cisplatin (Figure 13) combined with the WEE1 inhibitor adavosertib (AZD-1775, Figure 13, Table 3) in adult patients with metastatic TNBC, enrolling 34 participants. The study demonstrated modest antitumor activity, with an objective response rate of approximately 26% and a median progression-free survival of 4.9 months, not reaching the predefined efficacy threshold of ORR ≥ 30%. Treatment was associated with a notable incidence of adverse events.

## 6. Safety, Selectivity, and Translational Limitations

Despite the strong rationale for targeting the DDR in TNBC, the successful translation of these strategies into routine clinical practice remains challenging [98,99]. TNBC is molecularly heterogeneous, and only a subset of tumors harbor BRCA1/2 alterations, HRD, replication-stress-associated dependencies, or other DDR-related vulnerabilities that may confer sensitivity to DDR-targeted therapies. Consequently, therapeutic responses vary considerably among patients, limiting the broader applicability of synthetic lethality-based approaches [99,100].

One of the major limitations of DDR inhibition is the relatively narrow therapeutic window. Although TNBC cells frequently exhibit high levels of genomic instability and replication stress, many normal tissues, particularly the bone marrow and gastrointestinal epithelium, also rely on DDR pathways to maintain genomic integrity. As a result, hematological toxicities, gastrointestinal adverse events, and treatment-related fatigue remain common across multiple classes of DDR inhibitors [101]. These toxicities are consistent with the dependence of normal proliferating tissues on intact DDR and checkpoint pathways, and they become particularly relevant when DDR inhibitors are combined with chemotherapy, radiotherapy, or other DNA-damaging agents [36,102].

Another important challenge is achieving sufficient target selectivity. ATR belongs to the phosphatidylinositol 3-kinase-related kinase (PIKK) family, which also includes ATM and DNA-PK. Because these kinases share highly conserved ATP-binding domains, selective ATR inhibition without affecting related family members can be pharmacologically challenging. Off-target activity may increase toxicity and reduce the therapeutic window. Although next-generation ATR inhibitors such as ceralasertib, camonsertib, and elimusertib demonstrate improved selectivity profiles, anemia, thrombocytopenia, and neutropenia continue to represent common dose-limiting toxicities in clinical studies [99,103].

Similar challenges apply to WEE1 and CHK1 inhibitors. TNBC frequently harbors TP53 mutations and therefore relies heavily on the G2/M checkpoint for survival under conditions of replication stress and DNA damage. While this creates a compelling therapeutic opportunity, inhibition of these checkpoint regulators may also affect normal proliferating tissues [98,99]. Clinical studies with adavosertib have demonstrated encouraging antitumor activity in TNBC; however, treatment has been associated with significant rates of neutropenia, thrombocytopenia, diarrhea, and fatigue [98,103]. Consequently, ongoing drug development efforts focus on improving kinase selectivity and optimizing treatment schedules to maximize efficacy while minimizing toxicity.

For emerging targets such as POLQ, the primary translational challenge is the limited clinical experience currently available. Since Polθ expression is generally low in normal adult tissues but elevated in many HR-deficient and BRCA-mutated TNBCs, POLQ inhibition may offer a more favorable therapeutic window than checkpoint kinase inhibition [100]. Nevertheless, long-term safety data remain limited, and the predictive value of POLQ expression as a biomarker of therapeutic response requires prospective clinical validation [46].

Patient selection represents another major obstacle. Although germline BRCA1/2 mutations and genomic HRD scores have become established biomarkers for PARP inhibitor therapy, equivalent predictive biomarkers for ATR, WEE1, DNA-PK, or POLQ inhibitors have not yet been validated [99,100]. Moreover, genomic HRD signatures largely reflect historical DNA repair defects and may not accurately capture the current functional status of HR. This limitation is particularly relevant in TNBC, where restoration of HR through BRCA reversion mutations, 53BP1 loss, or replication fork stabilization may occur during treatment. Therefore, increasing attention is being directed toward functional biomarkers, including RAD51 foci formation, replication stress signatures, ATM loss, and circulating tumor DNA monitoring, although these approaches have not yet been standardized for routine clinical use [100]. Thus, biomarker maturity differs across DDR-targeted strategies: germline BRCA1/2 status is clinically established for PARP inhibitor selection in defined settings, genomic HRD scores provide context-dependent stratification, whereas RAD51 foci, replication-stress signatures, ATM loss, and ctDNA-based monitoring remain functional or dynamic biomarkers requiring further standardization [104,105,106].

To avoid overinterpreting preclinical or early clinical findings, Table 4 summarizes these strategies as a translational maturity framework rather than as a treatment algorithm. The table compares established therapies and emerging DDR-directed approaches relevant to TNBC or TNBC-overlapping biomarker-defined populations, with emphasis on evidence level, clinical positioning, and current limitations.

The development of combination therapies further increases translational complexity. Preclinical studies frequently demonstrate synergistic interactions between PARP inhibitors and agents targeting ATR, WEE1, DNA-PK, or immune checkpoint pathways [98,99]. However, overlapping toxicities often limit dose intensity in patients. Myelosuppression is particularly problematic when DDR inhibitors are combined with platinum-based chemotherapy or radiotherapy, both of which remain important components of TNBC treatment [101]. In addition, integration of DDR-targeted therapies with immunotherapy raises unresolved questions regarding optimal patient selection, treatment sequencing, immune-related toxicity, and schedule optimization. Although such combinations are mechanistically attractive, their clinical implementation may require intermittent dosing, sequential scheduling, or biomarker-enriched trial designs [50,62,96]. Overall, successful translation of DDR-targeted strategies in TNBC will depend less on target inhibition alone and more on achieving a clinically acceptable therapeutic index through biomarker-defined patient selection, rational combinations, and schedule optimization.

## 7. Future Directions and Limitations

The future development of DDR-targeted therapies in TNBC will depend on moving beyond static biomarker categories and single-agent synthetic lethality concepts toward dynamic, biomarker-guided, and combination-based therapeutic strategies [102]. Although PARP inhibitors and platinum-based chemotherapy have validated the clinical relevance of DNA repair vulnerabilities in BRCA1/2-mutant and HRD-enriched breast cancer, their efficacy is limited by primary and acquired resistance, interpatient heterogeneity, toxicity, and uncertainty regarding optimal sequencing and combination regimens [98,99,100]. In TNBC, pharmacological disruption of this pathway has been explored as an emerging strategy to increase DNA damage, impair repair adaptation, and sensitize tumors to DNA-damaging therapies [107].

### 7.1. Neddylation as an Emerging DDR-Proteostasis Vulnerability

In addition to direct targeting of DDR signaling pathways, modulation of post-translational regulatory mechanisms has emerged as an attractive therapeutic strategy in TNBC. The neddylation pathway is of particular interest because it controls the activity and turnover of numerous proteins involved in DNA repair, cell-cycle progression, and replication stress responses. Consequently, pharmacological inhibition of neddylation has the potential to enhance HRD-associated vulnerabilities and sensitize tumor cells to DNA-damaging therapies [107,108].

Protein neddylation is a highly conserved post-translational modification involving the covalent conjugation of the ubiquitin-like protein NEDD8 to target substrates, mainly leading to the activation of Cullin-RING ligases (CRLs) [109,110]. The process begins with the NEDD8-activating enzyme (NAE), which triggers a series of enzymatic reactions. These reactions lead to conformational shifts in cullin scaffolds, a necessary event for connecting ubiquitin-conjugating enzymes (E2s) with their target substrates [109,111,112]. The neddylation pathway regulates the activity of around 300 CRLs, influencing the degradation of almost 20% of all protein substrates targeted by the ubiquitin-proteasome system. This regulation is crucial for maintaining cellular protein balance (proteostasis) and controlling essential functions like DNA replication, cell division, and adaptation to stress. In malignant contexts, including TNBC, the pathway is frequently overactivated, providing a mechanistic rationale for its therapeutic inhibition [109,113].

The development of NAE inhibitors was based on the theory that inhibiting the neddylation pathway could heighten HRD by disturbing the proteostasis within DNA repair systems [109,111]. Mechanistic evidence indicates that neddylation plays a role in the dynamic recruitment of the BRCA1-BARD1-RAP80 complex to DSB sites [113,114]. As a result, blocking neddylation might elevate the level of DNA damage in tumor cells and interfere with the control of DNA repair proteins, ultimately intensifying HRD-related vulnerabilities and making BRCA1/2-mutant or HRD-positive cancers more responsive to DNA-damaging therapies [115,116]. The first-in-class NAE inhibitor pevonedistat reacts with NEDD8 within the NAE active site to form a stable covalent NEDD8-MLN4924 adduct [110,117]. While next-generation agents such as TAS4464 have demonstrated superior biochemical potency (IC_50_ of ~0.96 nM), their clinical development has been hampered by dose-limiting toxicities, including severe hepatotoxicity [118,119]. Alternatively, selective inhibition of the DCN1-UBE2M interaction with high-affinity small-molecules like DI-591 and DI-404 represents a more targeted approach, aiming to disrupt specific CRL functions, such as cullin 3 (CUL3) mediated nuclear factor erythroid 2-related factor 2 (NRF2) turnover, potentially providing a wider therapeutic window than pan-NAE inhibition [119,120].

Preclinical findings highlight the efficacy of neddylation inhibition within the context of TNBC. Both TNBC cell lines and patient-derived xenograft (PDX) models show a susceptibility to pevonedistat, which results in widespread DNA damage and growth arrest, independent of BRCA1 mutation presence [107,108]. This vulnerability is partially attributed to the overexpression of NAE1 in TNBC relative to other breast cancer subtypes, suggesting that NAE1 levels may serve as a predictive biomarker for treatment response [107]. Furthermore, pharmacological neddylation inhibition has been shown to induce a “BRCA-like” phenotype by impairing the recruitment of the BRCA1-BARD1-RAP80 complex to DSB sites [114]. Mechanistically, blocking neddylation may sensitize tumors to PARPis by both reducing HR repair efficiency and increasing PARP trapping through elevated replication stress [121]. The combination of pevonedistat with cisplatin has been reported to increase DNA damage and induce tumor shrinkage in preclinical TNBC models [107]. Pevonedistat has also been shown to enhance radiosensitivity in breast cancer cells by promoting G2/M arrest and impairing NHEJ-mediated repair [122].

### 7.2. Dynamic HRD Assessment and Resistance Monitoring

A major limitation of current DDR-targeted therapy is that static genomic biomarkers may not fully reflect the current DNA repair state of a tumor. In particular, BRCA1/2 mutation status or genomic HRD signatures may indicate historical defects in homologous recombination, but they may not capture functional restoration of HR during treatment [123]. Resistance may emerge through BRCA reversion mutations, partial HR restoration associated with 53BP1 or Shieldin complex loss, restoration of replication fork protection, altered drug efflux, or broader rewiring of DDR networks after exposure to PARP inhibitors, platinum agents, or emerging DDR-targeted drugs [106,124]. Understanding which synthetic lethal interactions are consistently effective across various genetic backgrounds, compared to those that are highly specific to certain contexts, will be crucial for stratifying patients. This includes exploring the roles of POLQ/TMEJ dependence, checkpoint adaptation, and the impact of neddylation on DDR proteostasis [103].

Thus, genomic HRD scores should be interpreted as static or historical genomic scar assays, whereas RAD51 foci formation and ctDNA-based monitoring represent more dynamic approaches that may better capture current HR function and emerging resistance [123,125]. Future therapeutic development should therefore move beyond a binary BRCA-mutated versus BRCA-wild-type framework toward a dynamic model of DNA repair capacity that integrates germline and somatic testing with functional HR biomarkers, replication-stress and fork-protection markers, and longitudinal monitoring of resistance evolution, including ctDNA-based approaches where supported by disease-specific evidence [102,106]. Immune consequences of DDR perturbation, including cGAS–STING-mediated signaling, may further inform rational combination strategies, but should not yet be presented as a validated biomarker for treatment selection in TNBC without additional direct evidence [126].

However, these approaches are not yet standardized for routine TNBC management. Their implementation will require prospective validation, harmonized assay protocols, reproducible scoring systems, clinically meaningful cutoffs, and evidence that biomarker-guided adaptation improves patient outcomes.

### 7.3. Next-Generation Drug Design and Medicinal Chemistry Opportunities

Future opportunities for medicinal chemistry include the development of next-generation PARP inhibitors, PARP1-selective agents, PARP1 degraders, and targeted protein degradation technologies [127,128]. PARP1-selective inhibitors and PARP1 degraders are being explored as potential strategies to preserve antitumor activity associated with PARP1 targeting while improving the therapeutic window, including by reducing toxicities linked to broader PARP1/2 inhibition. This rationale is supported by preclinical data for highly selective PARP1 inhibitors, but clinical confirmation of an improved therapeutic index remains necessary [128].

Targeted protein degradation technologies (TPD), including PROTACs and molecular glues, may overcome some limitations of reversible enzymatic inhibition by eliminating DDR proteins rather than transiently suppressing their catalytic activity [100]. In the DDR field, targeted protein degradation may be particularly attractive when catalytic inhibition is insufficient to suppress scaffold functions, target reactivation, or adaptive pathway rewiring. However, TPD strategies face important translational challenges, including high molecular weight, limited membrane permeability, suboptimal pharmacokinetic properties, dependence on ternary complex formation, variable expression of the recruited E3 ligase, potential resistance within the ubiquitin-proteasome system, and uncertainty regarding the long-term consequences of sustained target degradation [100].

Structure-guided optimization and artificial intelligence-assisted design may also support the development of more selective DDR modulators with improved potency, pharmacokinetic/pharmacodynamic properties, and scheduling compatibility [102,129]. Such approaches may be particularly useful for targets with narrow therapeutic windows, including ATR, CHK1, WEE1, and POLQ. However, AI-assisted approaches should be viewed as enabling tools for selectivity profiling, ADMET prediction, binding-mode analysis, and multiparameter optimization rather than as substitutes for experimental validation, mechanistic studies, and translational assessment [130].

### 7.4. Translational Priorities

Overall, the future success of DDR-targeted therapies in TNBC will depend not only on the identification of new synthetic lethal interactions but also on improvements in drug selectivity, toxicity management, biomarker development, and rational combination strategies. Addressing tumor heterogeneity, improving functional assessment of HRD, and overcoming acquired resistance mechanisms will be essential for translating preclinical findings into durable clinical benefit for patients with TNBC [98,99,100]. The most immediate translational priorities are prospective biomarker validation, optimization of therapeutic index, rational scheduling of combinations, and clinical trial designs that account for molecular heterogeneity and evolving resistance.

## Figures and Tables

**Table 1:** Molecular heterogeneity of TNBC and therapeutic implications.

TNBC Subtype	Key Features	Potential Vulnerabilities	Translational Status
Basal-like 1 (BL1)	High DDR activity and proliferative signaling	PARPi, ATRi, WEE1i	PARP inhibition is clinically relevant mainly in BRCA1/2-mutated or HRD-enriched settings; ATR/WEE1 targeting remains largely investigational
Basal-like 2 (BL2)	Growth factor signaling	EGFR/PI3K-directed approaches	Investigational or limited clinical benefit depending on target and biomarker selection
Mesenchymal	EMT-associated phenotype and invasive behavior	TGFβ, SRC pathways	Mainly preclinical or investigational
Luminal androgen receptor (LAR)	Androgen receptor dependence	AR-directed therapies	Clinical benefit observed in selected AR-positive cases; not a universal TNBC strategy
Immunomodulatory	Immune infiltration and inflammatory signaling	ICIs	Clinically relevant in selected biomarker-defined settings

DDR, DNA damage response; EMT, epithelial–mesenchymal transition; EGFR, epidermal growth factor receptor; PI3K, phosphoinositide 3-kinase; TGFβ, transforming growth factor beta; SRC, proto-oncogene tyrosine-protein kinase Src; PARPi, poly(ADP-ribose) polymerase inhibitors; ATRi, ATR inhibitors; WEE1i, WEE1 kinase inhibitors; ICIs, immune checkpoint inhibitors.

**Table 2:** Major predictive biomarkers and targeted therapeutic strategies in TNBC based on [24].

Biomarker/Target	Biological Role in TNBC	Targeted Therapy	Clinical Effectiveness
BRCA1/2 mutations	Defective homologous recombination DNA repair	PARP inhibitors (olaparib, talazoparib), platinum-based chemotherapy	Improved progression-free survival (PFS) and overall survival (OS) in BRCA-mutated TNBC patients
PD-L1 expression	Immune evasion through PD-1/PD-L1 signaling	Pembrolizumab, atezolizumab	Enhanced response rates and survival outcomes, particularly when combined with chemotherapy
Tumor-infiltrating lymphocytes (TILs)	Indicator of active antitumor immune response	Prognostic marker and potential indicator of immune responsiveness	Associated with improved disease-free survival (DFS), OS, and better response to immune checkpoint inhibitors
TROP-2	Cell-surface glycoprotein overexpressed in TNBC	Sacituzumab govitecan	Significant improvement in PFS and OS in metastatic TNBC
Androgen receptor (AR)	Driver of the luminal androgen receptor (LAR) subtype	Enzalutamide, bicalutamide	Clinical benefit observed in selected AR-positive TNBC patients; efficacy remains under investigation
EGFR overexpression	Promotes proliferation and tumor progression	Cetuximab, EGFR inhibitors	Limited clinical benefit; potential therapeutic option in selected patients
PI3K/AKT/mTOR pathway alterations	Cell growth, proliferation, and survival	AKT inhibitors (capivasertib, ipatasertib)	Activity in biomarker-selected TNBC populations
TP53 mutations	Loss of tumor suppressor function and genomic instability	No approved targeted therapy	Primarily prognostic marker associated with aggressive disease and poor outcomes
HER2-low expression	Low HER2 expression in a subset of TNBC	Trastuzumab deruxtecan	Emerging therapeutic option with encouraging clinical activity
EMT-related markers (e.g., TGF-β)	Invasion, metastasis, and treatment resistance	Experimental TGF-β inhibitors	Under clinical investigation; no established benefit yet

DFS, disease-free survival; EGFR, epidermal growth factor receptor; OS, overall survival; PFS, progression-free survival; TILs, tumor-infiltrating lymphocytes.

**Table 3:** Clinical Trials of PARP and WEE1 Inhibitors in TNBC.

Drugs Used (EN)	Phase	Title	Clinical Trial ID
AZD2281 (olaparib)	2	Phase II Study of AZD2281 in Patients with Known BRCA Mutation Status or Recurrent High Grade Ovarian Cancer or TNBC	NCT00679783
talazoparib	2	Phase II Trial of Talazoparib in BRCA1/2 Wild-type HER2-negative Breast Cancer and Other Solid Tumors	NCT02401347
cisplatin; veliparib	2	Cisplatin with or without Veliparib in Treating Patients with Recurrent or Metastatic TNBC and/or BRCA Mutation-Associated Breast Cancer	NCT02595905
cisplatin; rucaparib	2	PARP Inhibition for TNBC with BRCA1/2 Mutations	NCT01074970
paclitaxel; iniparib (SAR2405550, BSI-201)	2	Two Regimens of SAR240550/Weekly Paclitaxel and Paclitaxel Alone as Neoadjuvant Therapy in TNBC Patients	NCT01204125
BKM120 (buparlisib); BYL719 (alpelisib); olaparib	1	Phase I Study of the Oral PI3K Inhibitor BKM120 or BYL719 and the PARP Inhibitor Olaparib in Patients with Recurrent TNBC or Ovarian Cancer	NCT01623349
niraparib; pembrolizumab	1/2	Niraparib in Combination with Pembrolizumab in Patients with TNBC or Ovarian Cancer	NCT02657889
olaparib; durvalumab	2	Phase II Multicenter Study of Durvalumab and Olaparib in Platinum-Treated Advanced TNBC (DORA)	NCT03167619
fluzoparib; apatinib	1	Study of Fluzoparib in Combination with Apatinib in Ovarian or Breast Cancer Patients	NCT03075462
olaparib; radiation therapy	1	Olaparib and Radiation Therapy for Patients with TNBC	NCT03109080
AZD2281 (olaparib); carboplatin	1	AZD2281 Plus Carboplatin to Treat Breast and Ovarian Cancer	NCT01445418
olaparib; onalespib	1	Olaparib and Onalespib in Treating Patients with Solid Tumors That Are Metastatic or Cannot Be Removed by Surgery or Recurrent Ovarian, Fallopian Tube, Primary Peritoneal, or TNBC	NCT02898207
olaparib; durvalumab	1	Olaparib and Durvalumab in Treating Participants with Metastatic TNBC	NCT03544125
cisplatin, AZD-1775 (adavosertib)	2	CISPLATIN + AZD-1775 in Breast Cancer	NCT03012477

**Table 4:** Clinical positioning, level of evidence, and translational maturity of established and emerging therapeutic strategies relevant to TNBC or TNBC-overlapping biomarker-defined populations.

Therapy/ Target	Current Status	Role in TNBC	Biological Rationale	Preclinical Evidence	Clinical Evidence	Translational Maturity *
Platinum chemotherapy	Standard of care	First-line/neoadjuvant	Established	Extensive	Approved	High
PARPi	Approved	BRCA1/2-mutant and HER2-negative TNBC	Strong	Extensive	Approved	High
Immune checkpoint inhibitors	Approved	PD-L1-positive disease	Strong	Extensive	Approved	High
Sacituzumab govitecan	Approved	Second-line metastatic TNBC	Strong	Extensive	Approved	High
Trastuzumab deruxtecan	Approved	HER2-low TNBC	Strong	Extensive	Approved	High
ATR inhibitors	Phase I/II	PARPi-resistant HRD tumors	Strong	Extensive	Early clinical	Moderate
WEE1 inhibitors	Phase I/II	TP53-mutated TNBC	Strong	Extensive	Early clinical	Moderate
DNA-PK inhibitors	Early clinical development	Combination with radiotherapy or PARPi	Strong	Moderate	Limited	Low
POLQ inhibitors	Early clinical development	PARPi-resistant HRD tumors	Strong	Emerging	Early clinical	Low to Moderate
RAD51 inhibitors	Preclinical/Early translational	HR restoration-mediated PARPi resistance	Strong	Moderate	Minimal	Low
Neddylation inhibitors	Investigational	Sensitization to PARPi and DNA-damaging therapies	Emerging	Moderate	Limited	Low

* High: clinically validated and approved in defined settings; Moderate: supported by early clinical studies or strong translational rationale; Low: supported primarily by preclinical or early translational evidence. The descriptors “extensive,” “moderate,” and “limited” are qualitative summaries of the breadth and maturity of available evidence and should not be interpreted as formal evidence grading.

## Data Availability

No new data were created or analyzed in this study.

## Abbreviations

The following abbreviations are used in this manuscript:

53BP1	p53-binding protein 1
ABC	ATP-binding cassette
ABCC1	ATP-binding cassette subfamily C member 1
ABCG2	ATP-binding cassette subfamily G member 2
AKT	Protein kinase B/AKT serine-threonine kinase
alt-NHEJ	Alternative nonhomologous end-joining
ATM	Ataxia telangiectasia mutated
ATP	Adenosine triphosphate
ATR	Ataxia telangiectasia and Rad3-related
ATRi	ATR inhibitor
BARD1	BRCA1-associated RING domain protein 1
BCRP	Breast cancer resistance protein
BER	Base excision repair
BET	Bromodomain and extra-terminal
BRCA1	Breast cancer susceptibility gene 1
BRCA2	Breast cancer susceptibility gene 2
BRCT	BRCA1 C-terminal domain
BRD4	Bromodomain-containing protein 4
CDK	Cyclin-dependent kinase
cGAS	Cyclic GMP-AMP synthase
CHK	Checkpoint kinase
CRBN	Cereblon
CRL	Cullin–RING ligase
CST	CTC1-STN1-TEN1 complex
ctDNA	Circulating tumor DNA
CtIP	CtBP-interacting protein
CTRB	C-terminal RAD51-binding motif
CUL3	Cullin 3
DBD	DNA-binding domain
DDR	DNA damage response
DNA2	DNA replication helicase/nuclease 2
DSB	Double-strand break
DSS1	Deleted in split hand/split foot protein 1
dsDNA	Double-stranded DNA
D-loop	Displacement loop
E2s	Ubiquitin-conjugating enzymes
ER	Estrogen receptor
EXO1	Exonuclease 1
GPX4	Glutathione peroxidase 4
HDAC	Histone deacetylase
HER2	Human epidermal growth factor receptor 2
HLTF	Helicase-like transcription factor
HR	Homologous recombination
HRD	Homologous recombination deficiency
HRR	Homologous recombination repair
HSP90	Heat shock protein 90
IARC	International Agency for Research on Cancer
IRAK4	Interleukin-1 receptor-associated kinase 4
KB1P	BRCA-deficient mouse mammary tumor model KB1P
KB2P	BRCA-deficient mouse mammary tumor model KB2P
LIG3	DNA ligase III
LOH	Loss of heterozygosity
LST	Large-scale transitions
MDM2	Mouse double minute 2 homolog
MEK	Mitogen-activated protein kinase kinase
MDR1	Multidrug resistance protein 1/P-glycoprotein/ABCB1
MMR	Mismatch repair
MRN	MRE11–RAD50–NBS1 complex
NAE	NEDD8-activating enzyme
NEDD8	Neural precursor cell expressed developmentally downregulated protein 8
NER	Nucleotide excision repair
NES	Nuclear export signal
NHEJ	Non-homologous end joining
NLS	Nuclear localization signal
NRF2	Nuclear factor erythroid 2-related factor 2
OB fold	Oligonucleotide-binding fold
ORR	Objective response rate
PALB2	Partner and localizer of BRCA2
PAR	Poly(ADP-ribose)
PARG	Poly(ADP-ribose) glycohydrolase
PARP	Poly(ADP-ribose) polymerase
PARPi	PARP inhibitor
PAXIP1	PAX-interacting protein 1
p53	Tumor protein p53
pCR	Pathologic complete response
PDX	Patient-derived xenograft
PI3K	Phosphoinositide 3-kinase
PK/PD	Pharmacokinetic/pharmacodynamic
POI	Protein of interest
Polθ	Polymerase theta
POLQ	DNA polymerase theta gene
PR	Progesterone receptor
PROTAC	Proteolysis-targeting chimera
PTIP	Pax transactivation domain-interacting protein
PTEN	Phosphatase and tensin homolog
RAD51	RAD51 recombinase
RAS	Rat sarcoma viral oncogene homolog family
RCTs	Randomized controlled trials
RING	Really interesting new gene
ROS	Reactive oxygen species
RPA	Replication protein A
RS	Replication stress
SET8	SET domain-containing lysine methyltransferase 8
SHLD	Shieldin complex
SMARCAD1	SWI/SNF-related matrix-associated actin-dependent regulator of chromatin subfamily A containing DEAD/H box 1
ssDNA	Single-stranded DNA
STING	Stimulator of interferon genes
TAI	Telomeric allelic imbalance
TIM	TIMELESS circadian regulator / replication fork protection factor
TMEJ	Theta-mediated end joining
TNBC	Triple-negative breast cancer
TP53BP1	Tumor protein p53-binding protein 1
TPD	Targeted protein degradation
TR2	C-terminal RAD51-binding motif of BRCA2
UBA3	Ubiquitin-like modifier activating enzyme 3
UBE2M	Ubiquitin-conjugating enzyme E2 M
UFL1	UFM1-specific ligase 1
USP37	Ubiquitin-specific peptidase 37
VEGFR	Vascular endothelial growth factor receptor
WEE1	WEE1 G2 checkpoint kinase
WHO	World Health Organization
ZNF251	Zinc finger protein 251
